# Assessing Patient Discomfort in Smartphone-Based Teledentistry From the Perspective of Dental Professionals: Qualitative Interview Study

**DOI:** 10.2196/81163

**Published:** 2026-04-07

**Authors:** Margaret Shenouda, Boyen Huang, Emily C Schultz, Mohamed Estai, Sarbin Ranjitkar, Jeff P Louie, Patimaporn Pungchanchaikul

**Affiliations:** 1 School of Dentistry, University of Minnesota, 515 Delaware Street SE, Minneapolis, MN, 55455, United States, 1 612-624-7848; 2 College of Allied Health and Nursing, Minnesota State University, Mankato, MN, United States; 3 School of Human Sciences, University of Western Australia, Perth, Australia; 4 Adelaide Dental School, The University of Adelaide, Adelaide, Australia; 5 Department of Pediatrics, Medical School, University of Minnesota, Minneapolis, MN, United States; 6 Faculty of Dentistry, Khon Kaen University, Khon Kaen, Thailand

**Keywords:** mobile health, mHealth, smartphone, teledentistry, discomfort, patient, dental professional

## Abstract

**Background:**

Mobile health (mHealth) represents a modality of teledentistry that has the potential to improve access to dental care. Given that patient reactions to dental procedures can influence both clinician experience and care delivery, assessing patient discomfort when smartphones are used to capture dental images for teledentistry examinations is crucial.

**Objective:**

This study aimed to explore patient discomfort from the perspective of dental professionals using smartphone-based photography in teledentistry.

**Methods:**

A qualitative study was conducted through group interviews with a sample (N=10) of dental professionals, all of whom had experience capturing dental photos using smartphones equipped with an mHealth app at dental clinics and research facilities in Thailand and the United States. Audio-recorded interviews were transcribed, coded through consensus, and analyzed thematically.

**Results:**

The dental professionals, including dental specialists, general dentists, dental therapists, and dental students, reported minimal to no patient discomfort during smartphone-based dental photography. Key factors contributing to patient comfort during teledentistry encounters included clear communication, informed consent, and reassurances regarding privacy and data security.

**Conclusions:**

The findings suggest that providing patients with clear information and managing expectations can help reduce discomfort in teledentistry encounters. Improving communication strategies may enhance patient comfort, support the adoption of mHealth practices, and optimize interactions between patients and health care providers. Future research directions are indicated, such as directly assessing patient discomfort and identifying strategies to further minimize discomfort in teledentistry. Additionally, expanding teledentistry training in dental education and professional development will better equip dental professionals to effectively use this technology, ultimately improving accessibility and patient-centered care in dentistry.

## Introduction

Discomfort related to dental services can lead to dental care avoidance, impacting both oral and general health [1]. There are countless reasons why humans experience discomfort or distress, including camera shyness [2], a fear of being photographed [2], or concerns about their privacy or dignity being compromised [3]. Some people commonly experience anxiety or distress due to bleeding and/or pain caused by traumatic incidents [4]. Those emotional responses are different from dental anxiety, which is an anticipatory unpleasant feeling for dental procedures [1,5]. While evaluation scales varied among studies, meta-analyses estimated that up to 30% of children [6] and 15% of adults [7] experienced dental anxiety. Dental anxiety, fear, and phobia represent a spectrum of negative emotional reactions to dental environments and procedures [8], with “discomfort” often used as an umbrella term to describe experiences that range from mild unease to excruciating distress [9]. In this study, patient discomfort encompasses all levels of unpleasant feelings related to dental care.

Diagnostic tools, such as radiographs [5,10], intraoral cameras [11], and smartphones [12], make image assessments possible for dental professionals. Previous research has reported that pediatric patients experienced more anxiety when their dental treatment involved radiographs [5]. In adults, elevated anxiety biomarkers were also observed among those undergoing radiographic examinations for the first time [10]. To enhance patient experience and diagnostic accuracy, minimizing patient discomfort during the diagnostic imaging procedure is critical. A potential approach to lower dental discomfort is using mobile health (mHealth) technologies [13].

The mHealth practice provides a way for health care professionals to incorporate mobile phones and other technological devices into their practices [14]. As a modality of teledentistry [15], mHealth can be used for teleconsultation, telediagnosis, telemonitoring, telesupport, and teleintervention [16]. Smartphone-based dental photography also helps with communication and documentation in clinical settings [17]. The implementation of mHealth increases accessibility to health care services for rural and underserved communities [18]. The practice of mHealth grew in popularity during the COVID-19 pandemic when social distancing measures were required [19]. Previous research has reported that dental professionals [20,21], patients [21], and caregivers [22] all consider mHealth to be feasible and useful for remote dental services.

Teledentistry is increasingly being integrated into everyday dental procedures [23]. As mHealth technology seldom requires inserting instruments, devices, or radiographic film into the patient’s mouth, it is generally considered noninvasive. However, some patients have reported anxiety during video-based teledentistry consultations [24]. Earlier studies have demonstrated that most dental professionals could perceive patient discomfort in clinical settings [25] and also feel stressed managing patients with anxiety symptoms [26]. To the best of the authors’ knowledge, no existing literature has specifically addressed patient and clinician discomfort associated with photography-based teledentistry using mHealth approaches. Nonetheless, implementing strategies to reduce patient discomfort remains crucial for ensuring effective and patient-centered dental care [1], including teledentistry [24].

Whether mHealth practice would or would not induce discomfort in dental patients and/or dental professionals remained unclear. Therefore, this study addressed the following research questions: (1) whether dental professionals perceived potential discomfort from their patients in response to their use of the mHealth practice and (2) how patient discomfort (if any) influenced the dental professionals’ experience operating the smartphone and mHealth app to capture images of their patients’ teeth. Dental professionals’ perceptions were used as a proxy for patients’ reactions, as they were present during patient interactions, and interviewing patients in the clinical and research settings was not feasible. Thus, this study aimed to investigate dental professionals’ perspectives on patient discomfort when using a smartphone to capture images of patients’ teeth for teledentistry examinations. This study also explored the influence of patient discomfort on dental professionals’ photo-taking experience.

## Methods

### Overview

**Figure 1.**
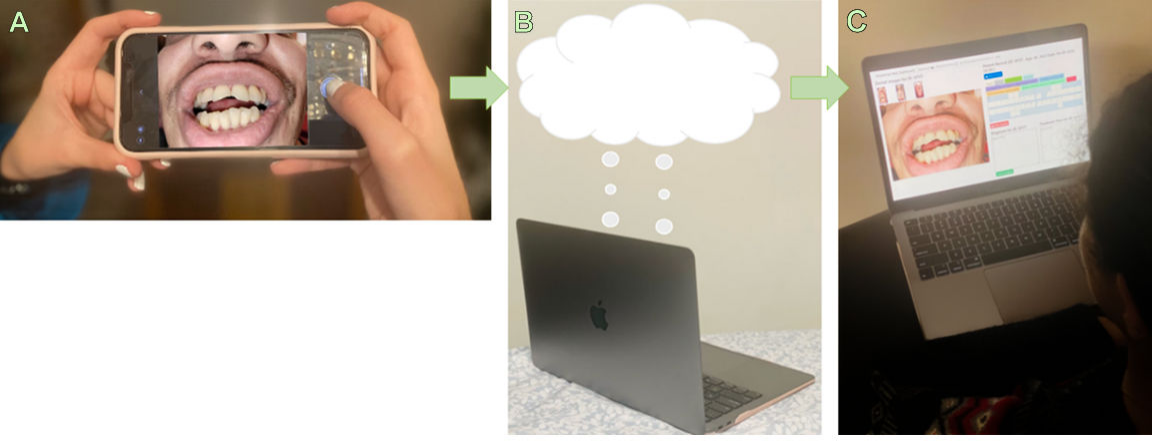
Illustration of the mobile health model: (A) a dental professional using the Teledental app to capture dental photos of a patient, (B) the data being uploaded from the smartphone to a secure cloud storage, and (C) a remote reviewer using the charting platform to review the dental photos asynchronously. The patient consented to have the images and data presented in the publication.

This qualitative study, forming a segment of a broader teledentistry research initiative, received approval from the University of Minnesota Institutional Review Board (STUDY00014736). The study was conducted across several sites in Minnesota, United States, and Khon Kaen, Thailand, including clinical facilities at the University of Minnesota, Khon Kaen University, and research venues at the Minnesota State Fair. In this paper, the term “patient” encompasses both dental patients recruited from clinics and fairgoers recruited at the State Fair research facilities, as both groups underwent the same teledentistry procedure. The quantitative and qualitative research methods used in the overarching project were recently reported [12,20]. In the quantitative segment, 11 trained and calibrated dental professionals used a Samsung smartphone equipped with an image acquisition app Teledental (CSIRO) to take dental photos (frontal, upper occlusal, and lower occlusal views) from consented dental patients and fairgoers and uploaded the images to a secure cloud storage for remote reviewers to assess [12]. Figure 1 illustrates the flow of the photographic acquisition and assessment in this mHealth model.

### Ethical Considerations

This study was approved by the University of Minnesota Institutional Review Board (STUDY00014736). Written informed consent was obtained from all participants prior to data collection. All study personnel complied with institutional review board–approved protocols governing data access, confidentiality, and privacy. Participating dental professionals based in the United States received compensation (dental therapist: $40 USD; dental student: $15 USD); Thailand-based dental professionals did not, due to university restrictions on out‑of‑country payments.

### Study Design

For the qualitative segment, we used a study design guided by the technology acceptance model (TAM) [27] and interpretivism [28] to examine the experiences of dental professionals and remote reviewers in using mHealth technology [20]. This work represents a portion of the qualitative segment and specifically explores patient discomfort from the perspective of dental professionals. Except for 1 dental professional (MS) who is an author of this paper, all 10 of the 11 dental professionals who captured dental images in the larger study were invited via email to participate in this qualitative study. All 10 professionals consented and attended a 60-minute interview conducted via Zoom (Zoom Communications). A combination of one-on-one and group interviews were conducted, with group size ranging from 1 to 6 interviewees, determined by their availability. Due to time zone differences, interviewees from the United States and Thailand were placed in separate groups. A total of 2 US-based researchers conducted the interviews in English, and all interviewees were proficient in English for the discussions. The study adhered to the Consolidated Criteria for Reporting Qualitative Research guidelines throughout its execution and reporting [29].

### Research Team and Reflexivity

Each one-on-one or group interview was conducted by 2 interviewers: MS, a female honors college graduate with a bachelor of arts degree, and ECS, a female dental hygiene faculty member with a master of science degree. BH, a male senior dental academic and principal investigator with doctor of philosophy and doctor of dental surgery degrees, observed all sessions. Before the first interview, the interviewers received training to adopt a neutral interviewing approach, ensuring interviewees’ perspectives were captured without bias. While most interviewees had no prior relationship with the interviewers, 3 were previously acquainted with an interviewer as dental professionals in a prior study led by the observer. The interviewers and observer introduced themselves first, and the objectives and details of this study were explained through the informed consent form and at the start of each interview.

### Data Collection

The interviews were attended exclusively by the interviewees and researchers. During the Zoom sessions, the interviewers took field notes to record nonverbal cues and additional observations. All interviews were audio-recorded and securely stored on a cloud-based server. Following each interview session, interviewees were emailed the complete study instrument and given 2 weeks to provide additional feedback. Data saturation, defined as the point at which no new information emerged across 3 consecutive sessions [30], was a criterion applied to the broader qualitative project involving a larger participant pool [20]. Nevertheless, to ensure a comprehensive and diverse range of perspectives, all 10 dental professionals were interviewed across 4 sessions, irrespective of whether data saturation had been reached. No interviews were repeated for the same dental professionals.

The interview instrument consisted of guided questions designed to explore the dental professionals’ perspectives. These questions were adapted from published resources [31,32] and rephrased to align with the context of this qualitative study and the specific mHealth technology used. A multidisciplinary panel of researchers, with expertise in qualitative study design, medical informatics, and dentistry, reviewed and finalized the interview questions to ensure their relevance and comprehensiveness. All dental professionals were asked 2 questions by the same interviewer. The first question was as follows: “How comfortable or uncomfortable did you feel about using the technology for dental trauma cases?” This question focused on the dental professionals’ own comfort levels when using the smartphone and mHealth app. The second question was as follows: “How might you have observed either potential distress or comfort from patients of the study?” The question was intended to capture the dental professionals’ perspectives on their patients’ comfort level. These 2 questions were part of a broader qualitative study [20] conducted, observed, and analyzed by the same research team. Although the larger study explored additional dimensions of mHealth use, only these 2 questions are reported here because they specifically addressed the anxious or emotional reactions experienced by dental professionals or patients during teledentistry procedures.

### Data Analysis

The qualitative data from the interviews were analyzed using a consensus coding process to summarize responses and synthesize the results, with a deductive approach [33-36]. Prior to conducting the interviews, the coding team met via Zoom to calibrate their approach, ensuring consistency in interpretation across transcripts. Audio-recorded interviews were transcribed and independently reviewed by the coding team. Throughout the analysis, memo writing was used to record coder reflections, track emerging themes, and document the rationale behind coding decisions, thereby enhancing transparency. Intercoder reliability was addressed through a negotiated agreement strategy: coders had established procedures for resolving discrepancies in advance, and when disagreements occurred, they revisited the transcript, discussed interpretations, and reached consensus. The principal investigator (BH), who observed all interview sessions, was available to provide input when needed to ensure consistency. A hybrid coding framework was applied, combining deductive codes derived from the research questions and the TAM [27] with inductive codes that captured novel insights arising directly from the data. This flexible approach allowed the team to systematically examine anticipated themes while incorporating unexpected findings that enriched understanding of participants’ experiences [33,37]. After all interviews were completed, the research team convened via Zoom to review the transcripts collectively, identify recurring themes, and extract supporting evidence, including descriptive terms and illustrative quotes from participants. The transcripts and findings were not shared with interviewees for review.

## Results

### Overview

A total of 4 interviews were conducted. All 10 dental professionals participated, 4 from the United States (coded U1-U4) and 6 from Thailand (coded T1-T6). Although U1 to U4 were initially intended to be interviewed together, scheduling constraints led to U1 and U4 being interviewed individually and U2 and U3 together, whereas T1 to T6 were all available simultaneously and, as affiliates of the same institution in Thailand, participated in a single group interview. Table 1 lists the interview sessions, professions, genders, and countries of the dental professionals.

The thematic analysis process, in which patterns or themes are identified within qualitative data revealed 2 main themes from the interviewees’ responses: professional familiarity and confidence with mHealth technology, and patient reactions to smartphone imaging in dental care (Table 2).

**Table 1.** Interview sessions, professions, genders, and countries of the interviewees; 2 one-on-one interviews (sessions 1 and 3) and 2 group interviews (sessions 2 and 4) were conducted, with the group arrangement determined by the interviewees’ time availability.

Interview session	Profession	Gender	Country	Interviewee code
1	Dental student	Male	United States	U1
2	Dental therapist	Female	United States	U2
2	Dental student	Male	United States	U3
3	Dental student	Female	United States	U4
4	General dentist	Female	Thailand	T1
4	General dentist	Female	Thailand	T2
4	General dentist	Female	Thailand	T3
4	General dentist	Male	Thailand	T4
4	General dentist	Male	Thailand	T5
4	Dental specialist	Male	Thailand	T6

**Table 2.** Main themes, definitions, subthemes (code words), and representative transcript excerpts that contributed to theme construction.

Main theme	Definition	Subtheme	Example from transcripts
Professional familiarity and confidence with mHealth technology	How dental professionals’ prior experience and daily use of mobile phones shaped their comfort and perceived ease in adopting smartphone-based imaging	Confident adoption due to everyday use	“Everyone seemed comfortable with...using the technology. We use mobile phone everyday all the time so we are comfortable with the technology.”
		Personal ease contrasted with anticipated challenges for others	“I think it was very easy for me. I think it was something that I felt very comfortable being able to use, taking the images, making sure the images came out well. I felt very comfortable doing that. I know it may be more challenging for people who are not as familiar with being able to use that technology.”
Patient reactions to smartphone imaging in dental care	How dental professionals perceived patients’ emotional responses to smartphone‑based imaging	Patients appeared at ease once informed	“Some patients were like ‘oh yeah, this is pretty cool,’ like it’s the next step in doing things through electronics. I thought I got a lot of patients that felt at ease with this.” “I feel study participants [felt okay] once they understand and we explain to them the reason and what we’re doing, and they’re aware of it.” “We did not notice any anxiety or discomfort; it is like a normal part of an examination and there is no pain when taking photographs. Because we asked the patients to sign the consent and they already gave consent prior to taking the photos, they did not notice any discomfort or anxiety from patients. Their regular dental [exam] is more invasive than taking the photos.”
		Patients expressed unease linked to privacy, intimacy, or cultural context	“I work with patients that are immigrants or potentially illegal immigrants and I think they get really nervous, even if you’re saying you’re not going to be in the photo at all, things like that.” “The mouth is a very intimate place for a lot of people. Letting people look in your mouth, especially if you have insecurities or are concerned about something or there’s something that you know is a problem but you’ve been putting it off for a variety of reasons is difficult.”

### Professional Familiarity and Confidence With mHealth Technology

A total of 9 out of 10 dental professionals reported feeling comfortable using the mHealth app and taking dental photos. Several dental professionals attributed this to their familiarity with smartphones, as U4 and T4 noted they use mobile phones daily. U4 also expressed ease with both the app and photography process. Factors such as having a newer phone (stated by T4) and proper lighting and retraction (stated by U1) contributed to the feasibility of the process. However, U2 mentioned challenges with consistency, requiring a refresher before using the app due to infrequent use.

Despite overall comfort, 3 dental professionals highlighted specific concerns. U2, based in the United States, described using a cell phone in a dental setting as “invasive,” particularly when working with privacy-conscious patients. This discomfort stemmed from introducing the phone and ensuring patients understood their data were secure, as stated by U2:

Whipping out a cell phone is kind of always uncomfortable. Especially with some of the patient types that I work with where they might be a little bit more concerned about privacy.

U2 also noted a lack of prior experience with intraoral photography. T4 emphasized the importance of restricting data access to the research team, while T3, another dental professional based in Thailand, worried about appearing less competent in front of the patient when relying on an app for diagnosis.

### Patient Reactions to Smartphone Imaging in Dental Care

The majority of dental professionals observed no noticeable discomfort among patients during photo-taking. T1 suggested that dentists can help patients become accustomed to photography as part of dental examinations by adopting the approach of “we take a photo and show it to them*.*” U2 noted observing minimal discomfort, describing patients as “fine” but acknowledged some initial hesitancy, especially among immigrant patients who might harbor concerns about privacy or identification. U2, a US-based dental professional, noted that immigrant patients sometimes fear being photographed, even when assured they won’t appear in the image (see Table 2).

Despite this, U2 mentioned that some patients viewed technology integration as a necessary advancement. U3, acting as a research recruiter at the Minnesota State Fair, observed that patients were generally comfortable by the time they arrived, perhaps because they volunteered or were not in a dental facility for dental care. However, recruiting participants posed a challenge, echoing common issues with dental appointment attendance. U4 found that providing more detailed information and setting clear expectations reduced patient discomfort, making them “less anxious” and “more willing” to participate.

Dental professionals also speculated why patient discomfort was minimal. T1 noted that patients in emergency trauma situations were generally uncooperative and preferred faster tools such as intraoral cameras. Conversely, patients with nonemergency issues were described as “very comfortable.” T2 and T4 believed that obtaining informed consent and explaining the photo-taking process alleviated patient concerns. They emphasized that the procedure was painless and less invasive than standard dental examinations. T4 noted that patients seemed comfortable, particularly when dental photos were shared with them afterward to facilitate treatment discussions.

Clear communication was a recurring theme in reducing patient discomfort. T4 and U1 highlighted the importance of explaining the exclusion of identifiable features in photos. U1 stated the following:

Once we explained that [photos] wouldn’t be of any identifiable features, most patients were comfortable.

Transparency about the process helped foster trust and alleviate initial apprehension.

## Discussion

### Principal Findings

The overall consensus from dental professionals was that patients did not experience discomfort during smartphone-based dental photography. Although the telesupport function of some mHealth technologies could reduce patient discomfort [13], this study demonstrated that the asynchronous telediagnosis function of the mHealth technology could also minimize patient discomfort. Of further note, a prior study has reported a low level of dental patient anxiety from synchronous video consultation [24]. Thus, both synchronous and asynchronous teledentistry can decrease patient discomfort when dental professionals use these practices to communicate and examine patients.

One of the reasons for this finding is likely to include the physical safety characteristics of the mHealth model. Earlier studies have suggested that the use of X-rays in dental treatment increased patient anxiety, most likely due to the procedure’s uncomfortable nature [5], apprehension toward radiation exposure [5], and the perceived lack of control during image acquisition [10]. Compared to X-rays, taking photos using a smartphone does not inflict any pain and is not invasive. Hence, patients become less uncomfortable when receiving a teledentistry procedure than undergoing a radiographic examination. Another possible reason patients did not express discomfort may have been the communication provided by dental professionals before the teledentistry procedure. Dental professionals informed patients about what the dental photos would include and how they would be used, which likely contributed to patient comfort. This aligns with the recommendation from literature, which emphasizes obtaining clear informed consent and ensuring confidentiality, particularly regarding who would have access to recorded materials and how they would be used, can enhance patient comfort [38] and support the effectiveness of psychotherapy [2]. Furthermore, dental professionals could show patients the images of their teeth after photography to help strengthen patient trust. These highlight the importance of health care provider–patient communication and carry significant clinical implications for mHealth and teledentistry.

Beyond the clinical context, the findings of this study can also be interpreted through the lens of informatics and digital health system design. The TAM provided a useful framework for understanding how familiarity with smartphones influenced dental professionals’ confidence and ease of adoption [27]. From a systems perspective, the mHealth model used here illustrates key principles of human-technology interaction: usability [39], transparency [40], and trust [40]. Prior informatics reviews have emphasized that clear communication of data flows and privacy safeguards is essential for user acceptance of digital health tools [40,41]. Integrating these insights into system design suggests that teledentistry platforms should prioritize intuitive interfaces [40], explicit consent protocols [41], and secure data management to enhance both health care provider and patient comfort [40,41]. In this way, the study contributes not only to clinical practice but also to broader discussions on how digital health systems can be designed to balance efficiency, accessibility, and patient-centered care.

In teledentistry procedures, smartphones and intraoral cameras are often used as alternatives to each other. A recent study found that patients reported greater comfort with smartphone-based teledentistry compared to intraoral cameras, although smartphone photography required more time to complete a similar procedure [42]. Using a smartphone for diagnostics may cause less discomfort than an intraoral camera because smartphones are familiar devices used daily by many people. In contrast, intraoral cameras are only found in clinical settings and can feel more intrusive. In cases of traumatic dental injuries, high-quality photographs during the initial visit are essential for long-term follow-up, which may be required for up to 5 years [43]. Patients will need to undergo photography during most follow-up visits. As mHealth apps on smartphones feel less intimidating than intraoral photography, they could help reduce patient discomfort and encourage regular checkups.

Only 1 interviewee raised a unique concern regarding the potential discomfort that smartphone-based photography might cause among immigrant patients. Although all patient photos were deidentified during the research process and patients were reassured about privacy and confidentiality protections, maintaining anonymity in photography-based research involving migrant minority groups remains an important consideration [44]. To address this, patient education programs could help reassure patients about the safety of teledentistry, while professional development for clinicians and administrators could reinforce best practices for protecting patient privacy. Additionally, dental photographs captured through mHealth approaches should be securely integrated into electronic health records to ensure confidential access and long-term storage. For patients who are uncomfortable with teledentistry, in-person appointments should remain an option to ensure continued access to dental care.

Dental professionals also found the mHealth teledentistry model to be feasible, reporting a high level of comfort with its use. This aligns with findings in medical education, where students with prior exposure to telemedicine tend to feel more confident using such technologies [45]. On the basis of these insights, we recommend integrating comprehensive teledentistry training into dental education and ongoing professional development. These programs should prioritize building core competencies in teledentistry, such as obtaining informed consent and maintaining patient confidentiality during virtual encounters. Incorporating teledentistry training into dental curricula and clinical protocols could enhance practitioner readiness and improve patient care in remote settings. Standardizing communication protocols and ethical practices, such as consent and confidentiality, can help establish trust and ensure quality care. As teledentistry becomes more widespread, these educational and policy shifts will be essential for ensuring its effective and responsible implementation in clinical practice.

The interpretation of this study’s findings is subject to certain limitations. First, the small sample size of interviewees may restrict the ability to draw generalized conclusions, as a larger cohort might reveal additional perspectives. The pool of participants, consisting of 4 US-based and 6 Thailand-based dental professionals, may have introduced biases shaped by their unique clinical experiences, cultural backgrounds, patient populations, and photo-capturing environments. For instance, dental professionals assigned to the Minnesota State Fair research facilities were unlikely to encounter patients with emergency trauma cases, as such patients would typically seek care in clinics or hospitals rather than visiting the State Fair. Conversely, those working in clinical settings were more likely to manage patients presenting with pain, bleeding, or injuries and may have been more confident and experienced in clinical dentistry. Notably, privacy concerns among immigrant patients were raised by American dental professionals, whereas Thai dental professionals reported image concerns about appearing less competent in front of patients. These differences may reflect broader cultural expectations. An earlier study suggested that Thais were inclined to accommodate audience expectations, while Americans often balanced personal goals with audience interests [46]. Such cultural norms likely shaped how dental professionals perceived patient concerns and articulated their views. Given these contextual influences and the small, convenience-based sample, the transferability of findings is limited. Future research should purposely recruit larger and more diverse cohorts of dental professionals working with similar mHealth models to strengthen the depth, cultural breadth, and applicability of insights.

Another limitation of this study is its focus on patient discomfort as perceived by dental professionals, rather than directly assessing patient discomfort from the patients’ perspectives. Given the small sample size and the varied clinical experiences of the interviewees, interpretations of patient discomfort may reflect biases stemming from dental professionals’ subjective perceptions. While indirect assessments of patient discomfort from clinicians’ viewpoints may not capture the full scope and intensity of patients’ experiences, dentists are well positioned to observe and interpret discomfort [25], and they can also be affected by their patients’ discomfort [26]. Therefore, investigating patient discomfort from dental professionals’ perspectives remains a practical and informative approach, despite its limitations.

Future directions based on this study include exploring patient discomfort from the perspectives of both patients and caregivers, as well as identifying the factors contributing to discomfort associated with different teledentistry technologies. Additionally, developing and evaluating effective strategies to alleviate such discomfort is a critical area for future research. This study also underscores the importance of defining and cultivating core competencies in teledentistry, particularly in understanding and managing patient discomfort. Refining training curricula for current and future clinicians to incorporate these competencies will better prepare them to address patient concerns and maximize the effectiveness of teledentistry practices.

### Conclusions

This study highlights the feasibility of smartphone-based photography for teledentistry, with dental professionals expressing comfort in using the mHealth app. Familiarity with smartphones and clear communication helped facilitate the process, although minor challenges such as maintaining consistency in app use and concerns about smartphone introduction in clinical settings were noted. Patients exhibited minimal discomfort, likely due to the noninvasive nature of smartphone photography and the role of informed consent in setting expectations.

Clinically, integrating smartphone-based photography into teledentistry requires clear communication strategies to enhance patient comfort and trust. Future research should directly assess patient discomfort during teledentistry encounters and explore strategies to further minimize discomfort. Expanding teledentistry training in dental education and professional development will better equip dental professionals to use this technology effectively, ultimately improving accessibility and patient-centered care in dentistry.

## Supplementary material

10.2196/81163 Checklist 1 COREQ checklist.

## Abbreviations

mHealth: mobile health
TAM: technology acceptance model
